# Improve Integration of In Vitro Biofilm Body of Knowledge to Support Clinical Breakthroughs in Surgical Site Infection

**DOI:** 10.5435/JAAOSGlobal-D-20-00217

**Published:** 2021-11-04

**Authors:** Stuart Irwin, Brett Wagner Mackenzie, Brya G Matthews, Dustin L Williams, Jillian Cornish, Simon Swift

**Affiliations:** Department of Medicine (Irwin and Dr. Cornish), Department of Surgery (Dr. Wagner Mackenzie), and Department of Molecular Medicine and Pathology (Dr. Matthews and Dr. Swift), The University of Auckland, Auckland, New Zealand; (Dr. Williams); Department of Orthopaedics, University of Utah, Salt Lake City, UT (Dr. Williams), Department of Pathology (Dr. Williams), Department of Bioengineering (Dr. Williams), University of Utah, Salt Lake City, UT; and Department of Physical Medicine and Rehabilitation, Uniformed Services University, Bethesda, MD (Dr. Williams).

## Abstract

Prosthetics increase the risk of deep surgical site infections in procedures intended to restore function. In orthopaedics, prosthetic joint infections can lead to repetitive surgeries, amputation, or worse. Biofilm formation both in vitro and in vivo involves stages of attachment, accumulation, and maturation. The level of maturation affects susceptibility to antibiotics, the immune system, and the success of surgical interventions. A review of the literature indicates that orthopedic publications are less likely to mention biofilm. We have reviewed animal models of infection to assess in vivo models of prosthetic infection. Although most prosthetic infections seem to originate from local skin microbiota, clinically representative biofilm inocula are unusual. Biofilm-related end points are more widely adopted, but studies rarely include both quantification of adherent microbial burden and imaging of the in vivo biofilm. Failure to differentiate between planktonic and biofilm infections can skew research away from needed chronic disease models. In this review, we address prosthetic joint infections as an important model for chronic biofilm infection research, identify critical requirements for in vivo models of chronic infection, and propose that resistance to the terminology of biofilm research exists within both research and regulation, which could limit progress toward important orthopaedic targets.

Regenerative medicine attempts to restore tissue and organ function through repair or replacement of anatomy and plays a role in many specialties, including orthopaedics.^1^ To this end, advances in engineered biologics and complex implantable technologies promise notable improvements to human’s quality of life.^2^ An ageing population has driven increased demand for abiotic devices and devitalized tissue products and research into the infections associated with these implantable technologies (Figure 1, A).^3–5^ Increased use of implantable technology has amplified the risk of deep surgical site infections (dSSIs) because of the absence of on-board immune function and favorable biofilm accumulation conditions.^6,7^ Biofilm infections on hardware can develop resistance to antibiotics by leveraging their extensive antimicrobial tolerance, and persister cells within the biofilm can drive the reinfection cycle. High morbidity infections often become chronic, conferring substantial costs onto healthcare systems.^8,9^ These dSSIs have a devastating effect on quality of life of the patient, with 12% of patients describing the condition as equivalent to or worse than death.^10^

**Figure 1:**
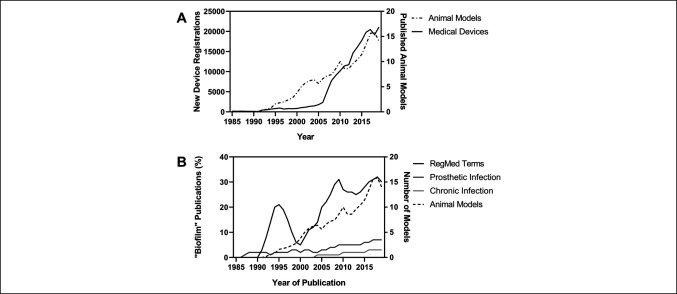
Graphs showing the use of biofilm in scientific articles using different terminologies for infection. **A**, The 5-year rolling averages of number of new devices registered with the Food and Drug Administration (FDA) each year between 1980 and 2000 (total = 2,47,192) versus total number of published animal models of prosthesis infection in Medical Subjects Heading (MeSH) Index for same period (total = 215). **B**, The 5-year rolling averages of the use of expanded Regenerative Medicine terminology (device, hardware, or implant and related or associated and infection; n = 3187) or orthopaedic terminology (periprosthetic, prosthetic related, and prosthetic associated; n = 13,433) found with the term biofilm in titles and abstracts over the period 1980 to 2000. Most of the manuscripts using the term biofilm in context with infections currently believed to be biofilms that are not found with terms typically used in orthopaedics. The term chronic infection was plotted for comparison as a historically consistent term, now believed to be primarily biofilm based when nonviral (n = 12,7442).

In orthopaedics, prosthetic joint infections (PJI) are known as the most intractable problem in the field. PJI exemplifies a worst-case outcome for an otherwise successful restorative surgical procedure, where a dSSI becomes irreversibly associated with the material used to restore joint function.^11^ These conditions are sufficiently serious that surgical intervention is typically required.^12,13^ Current studies estimated that 1% to 2% of total knee replacements will develop PJI, and this number is rising despite widespread use of well-established infection prevention measures.^14,15^ Mortality is as high as 7% with *Staphylococcus aureus* infections after surgical intervention.^10^ According to a recent review by Dlaska et al, a comprehensive investigation into the cause of surgical infections is cost prohibitive, and any successful findings would have limited applicability and require an additional decade to influence in clinical practice. Most importantly, any clinical trial involving dSSIs would be fraught with risk to patients, making clinically relevant animal treatment models a critical target for developing treatment strategies.^5^

We observe that translational research addressing treatment of orthopaedic infection is inhibited by dissonant cross-functional terminology, enabling inappropriate disease models and blunting regulatory controls. In this review, we evaluate the current understanding of biofilm-associated hardware infections in the context of PJI. Our aim was to propose tools for researchers and clinicians to advance our understanding of these recalcitrant infections. To this end, we provide a summary of the history of biofilm infections and evaluate inconsistencies between clinical and research usages of biofilm terminology. We assess in vivo models used in the search for interventions to address biofilm-based hardware infections and offer clinically relevant terminology that can be applied in both research and clinical settings.

## A History of Biofilm Infection

Biofilm is the natural state of most bacteria, and biofilm-based infections are not new to complex life on earth. Evidence of deep infection can be observed in Jurassic fossils,^16^ and many examples are found in the human archaeological records.^17^ Brain surgery undertaken to treat the pain of osteomyelitis was quite widespread and surprisingly survivable as early as the Neolithic period^18^; however, the dSSIs that often resulted from these trepanning procedures were not.^19^ Millennia later, Sir Alexander Fleming documented dramatic infections associated with trench warfare during World War I by demonstrating the clinical challenge presented by these septic combat wounds. He created an artificial wound in a cracked glass bioreactor to simulate shrapnel, wherein he cultured bacterial strains obtained from infected soldiers. These strains tolerated clinical antiseptics at highly cytotoxic concentrations for up to 24 hours,^20^ prophesizing the modern balance between antimicrobial treatment and tissue repair in entrenched infections^21^ and, eventually, the complex synergies of tolerance, resistance, and persistence in chronic infection.^8^ From then, Fleming undertook research that ultimately led to the use of penicillin in 1943 as the first scalable production antibiotic.^22,23^ That same year, curiously persistent subpopulations of bacteria were identified in chronic wounds,^24,25^ indicating that the battle against infection was not won, well before the relationship between antimicrobial resistance and tolerance in biofilm-laden infections was described. Twelve more antibiotic classes were discovered from 1938 to 1968, but only three additional classes were added since. Although clinical biofilm infection can be due to many environmental pathogens, *S aureus* is most often the cause^23^ and is now reemerging around the world as a major threat to human health.^26^ With ever-growing antibiotic resistance and declining treatment options, growing anxiety is observed in the medical community and regulatory bodies about the future of antibiotics for treating chronic infections.^27^

Although antibiotic development and discovery were waning, steps were being taken to prevent and treat dSSIs in elective surgery. Sir John Charnley reduced the incidence of dSSIs in his prosthetic hip surgeries using a filtered air environment,^28^ and by the 1970s, Buchholz and Engelbrecht^29^ improved outcomes of PJIs with the addition of antibiotics directly to bone cement during revision surgery. Shortly afterward, Bill Costerton connected his observations of environmental biofilm to internal medicine,^30^ and by the 1980s, biofilm was entering the medical vernacular.^31^ In 1987, a study by three separate laboratories using matched strains of *Candida albicans* D-1079 found more than a 50,000× difference between the minimum inhibitory concentrations (MICs) of established antifungals depending on the growth conditions used.^32^ Later studies indicated that the differences resulted from comparing MICs of planktonic cells to antifungal-tolerant biofilm.^33^ Given that MIC values alone determined clinical antibiotic treatment, this elicited a rethinking of the existing methodologies. As a result, the National Committee for Clinical Laboratory Standards introduced stringent growth controls for in vitro microbial susceptibility assays in 1993 in an attempt to standardize conclusions drawn from MIC assays.^34^ Although this was a critical advancement in vitro, variable tolerance in vivo remained a problem. For example, the antibiotic vancomycin is a common choice for musculoskeletal staphylococcal infections. It has an MIC for sensitive organisms of approximately 1 µg/mL^35^ and cytotoxicity to bone and tissue well above 1 mg/mL.^36^ Despite this 1000x˙ range, clinicians are known to apply vancomycin powder directly onto sensitive infected tissue in extreme cases.^37,38^ This implies that microbial tolerance to vancomycin in these likely biofilm cases exceeds its cytotoxicity, and clinicians must balance causing tissue damage against decreasing microbial bioburden, possibly with little success. ^38,39,40^ In point of fact, we are unaware of how a clinician could request evaluation of the biofilm equivalent of MIC, the minimum biofilm eradication concentration (MBEC), of vancomycin or any other antibiotic for a patient in distress.^41^ A paucity of standards, regulation, and guidance for the diagnosis and treatment of biofilm-related infection must be addressed concurrent to ongoing research.^42,43^

With mainstream adoption of the concept that biofilm formation is the defining characteristic of chronic infection, the struggle to adequately define the term biofilm sharpened. Unreliable MIC information made it apparent that the gap between clinical microbiology and the reality of infection was wide and not well understood. In 2010, Springer Publishing released a book titled “Biofilm Infections,” which included an exhaustive list of in vivo models of chronic infection.^44^ Two years later, Williams and Costerton^45^ proposed that the use of planktonic bacteria to model infection in vivo may be a limiting approach to the study of chronic infection, quietly calling into question all but three of the models reviewed in 2010. Caution is warranted when disruptive concepts enter medical discourse; however, the term “prudence of the lowest order”^46^ may be appropriate here, if translation of biofilm science to clinical practice has lagged in development because of this caution. We contend that the clinical dogma of biofilm and planktonic bacteria being interchangeable scientifically or that these infections are binary in nature is profoundly misleading in infection research and must be challenged more loudly. By understanding the history of clinically relevant biofilm in both scientific and clinical contexts, we can provide a foundation to design more relevant and functional studies in the absence of clear definitions and regulatory guidance.

## Biofilm Terminology and Definition

The term biofilm is often used to refer binary phenotypes of planktonic and biofilm existences of microorganisms. Planktonic bacteria are freely living and well represented by the laboratory broth culture. Biofilm bacteria, by contrast, are complex, diverse assemblages of bacteria that develop and maintain a privileged microecosystem and display a range of behaviors that clearly delineate them from pure broth culture bacteria.^44,47,48^ Because biofilm assemblages are nonuniform, the terminology applied to describe biofilms is variable, and a consensus definition has not been reached. This is further complicated by the implied specificity of the term, when in fact, this is how most bacteria live, and planktonic colony forming units (CFUs) that infection research is built on are the exception and not the rule. In this review, we consider biofilm under the broadest of definitions: an organized accumulation of bacteria.

**Figure 2:**
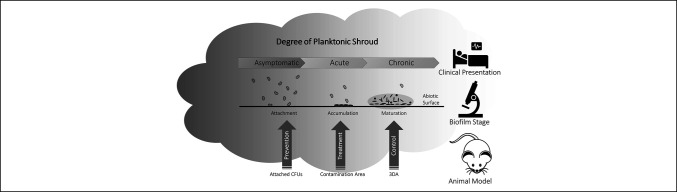
Illustration showing planktonic shroud in transition from acute to chronic infection. The relationship between the stages of biofilm (attachment, accumulation, and maturation), and typical clinical presentations (asymptomatic, acute, and chronic), and suggested disease models (nonrevision) for each (prevention, treatment, and control) that can provide the clear and relevant end points (attached colony forming units [CFUs], contamination, and three-dimensional aggregation [3DA]). Planktonic shroud is provided to illustrate its expected level in each model. Initial attachment may require planktonic inoculation, whereas CFU enumeration of a mature biofilm will be confounded by the presence of planktonic bacteria. Stages are cumulative; thus, a chronic latent infection may have both early acute and asymptomatic aspects, whereas asymptomatic is unlikely to have early acute or chronic latent aspects.

Biofilms form in stages, which can take as little as 24 hours in vitro^49,50^ but may continue to mature for days.^51^ Mature biofilm will often require weeks to develop in vivo, which is prohibitively slow in an animal model, but necessary to model chronic conditions.^52^ These models are difficult but yield highly applicable outcomes, as recently demonstrated in a 56-day open fracture model of subclinical infection demonstrating the presence of biofilm and low CFU inocula.^53^ Often, the presence of infection is used interchangeably as evidence of biofilm, and although this may be necessary clinically likely,^54^ the burden of proof in an animal model is higher. A clear understanding of the biofilm phenotype and maturation stage, how it maps to clinical presentation, and the consistent use of terminology critically informs the modeling of chronic infections, reproducibility, and translation of effective interventions. Categorization of the in vivo biofilm into meaningful terms that map to well-reviewed life cycle stages guides experimental design and supports translation of findings.^55,56^ The minimalist triad of attachment, accumulation, and maturation attributed to the study by Nishitani et al accomplishes this effectively^57^ and can be mapped effectively to prevention, treatment, and control strategies (Figure 2) detailed further. First, the attachment phase of a biofilm infection is often preceded by the development of a local acute infection or planktonic shroud and is where biological prevention strategies are focused, but both may be present.^58^ This initial attachment is often applied in animal models by inoculation with laboratory-grown planktonic CFUs. It is important to note that this is not the typical etiology of biofilm infections and may deviate from clinical relevance depending on the need for persistence in a given study.^59,60^ The aggregation of attached bacterial colonies characterizes the accumulation phase, whereby microcolonies capable of resisting phagocytosis and undergoing further expansion across the surface develop. The onset of the accumulation phase indicates failure of prevention because the infection evades control by the innate immune system.^61^ We propose that the accumulation phase represents the typical target for biological intervention strategies because symptoms become apparent. Delaying the onset of biofilm maturation and maintaining the biofilm in earlier, more vulnerable stages of development allow clinicians to strike a balance between the body's wound healing process and control of biofilm maturation. In animal studies, end points including biofilm coverage area on an implant or number of CFUs per unit area are most applicable and representative of this stage of clinical biofilm development. The maturation phase is characterized by vertical development of the biofilm, typically visible as three-dimensional structures under confocal or electron microscopy. Transition of the biofilm to the maturation stage represents a chronic, well-established infection that mirrors clinical presentation of dSSIs recalcitrant to treatment and is limited to either control strategies or surgical removal. Increased biofilm depth facilitates gradients of pH, O_2_, and nutrients, generating persister cells and establishing the intractable infection. These three developmental stages can be used to guide in vitro and in vivo research.

In addition to attachment, accumulation, and maturation, most descriptions of the infectious biofilm life cycle include a dispersal stage. Dispersal is the coordinated release of large numbers of infectious particles from a biofilm, often attributed to quorum sensing phenomena.^62^ Although dispersal can be demonstrated in vivo with dispersing agents, it is typically not observed under normal, survivable infectious conditions beyond minor shedding of infectious particles.^63^ Dispersal is an important maturation event but may not be critical for in vivo models of chronic infection.

Finally, an effective in vivo model of culture-negative prosthetic infection must minimize the amount of confounding acute infection present because persister cells drive the reinfection cycle. Current ex vivo culture techniques do not enable quantification of contributions to bioburden by planktonic versus biofilm lineages; however, attributing CFUs to chronic versus acute infection is inaccurate.

## Current Applications of Biofilm in Literature

We believe that the conflation of biofilm with clinical infection as a nonbinary condition is indicative of lapsed adoption of contemporary terminology in some areas of infection research. All biofilms are infections, but not all infections are biofilm, and biofilm comes in many forms. The work by Chomsky and Herman in Manufacturing Consent indicated that adoption or rejection of specific language can, among other things, lead to self-censorship and affect the communication and beliefs of entire groups with real-world effect,^64,65^ such as research design and clinical practice. The rate of adoption of terminology associated with biofilm was used in this review to indicate awareness and/or acceptance of the field of biofilm infection and its applicability to medical research. We searched PubMed for biofilm in titles and abstracts of publications about infection between 1980 and 2020. Three different terminology sets for infection were assessed. Orthopaedic terms prosthetic-related or prosthetic-associated, or periprosthetic infection were rarely used in combination with biofilm (Figure 1, B). Similarly, articles about chronic infection, which is a more general clinical term for infections now accepted generally to involve biofilm when referring to nonviral infections, referred to biofilm in <5% of their abstracts even in the past decade. Finally, articles about infection related to hardware, devices, or implants (search terms: hardware/device/implant [hyphen] associated/related AND infection), terms that began to emerge in the 1990s as regenerative medicine were adopted (RegMed terms), showed much faster adoption and higher use of the term biofilm (95% CI, of slope = 0.79 to 1.14 versus 0.15 to 0.20 increase in percentage of publications per year, Figure 1, B). These terms refer to a growing range of implantable technologies beyond orthopaedics. This implies that the regenerative medicine researchers were either more aware of or accepting of the underlying science of biofilm infection than other researchers. We believe that the inconsistent application of biofilm terminology is scientifically limiting and affects progress toward addressing orthopaedic infections.

## Typical Prosthetic Joint Infection Pathology

Chronic hardware infections are not typically generated by environmental contaminants in surgical suites, nor by hematologic transfer in healthy subjects. Rather, endogenous transfer of staphylococcal biofilm during surgery from deep pores or hair follicles colonized with biofilm that cannot effectively be decontaminated is the likely cause.^11,66^ Preformed biofilm can immediately establish on damaged and devitalized tissue or directly on the abiotic surface of a prosthesis, carrying with it antibiotic tolerance. During the acute postoperative period, this infection typically establishes on permissive tissue or sequestra and transfers to the prosthetic joint surface as it matures, and this is important to consider when modeling infection.^48,67,68,69,70,71^

PJI generates a highly acidic, nutrient-starved, anoxic environment whereby the defensive biofilm matrix confers protection from immune cells, chemicals, and antimicrobial proteins of the host immune system.^51,72,73,74,75^ Even in early-stage biofilm development, microcolonies consisting of relatively few cells are capable of generating a sufficiently acidic environment that degrades bone, can cause hardware loosening, and exceeds the phagocytic capacity of first-responder neutrophils.^76,77^ Persister cells are found at the deepest levels of the mature biofilm fortress and drive the reinfection cycle.^78–80^ The quiescent phenotype of persister cells results in extreme tolerance to antibiotics, allowing the organism to persist and eventually develop heritable genetic resistance.^8,81^ For these reasons, persister cells should be present in any credible model of mature biofilm addressing chronic infection.

It is important to consider that clinical biofilm stages inform critical treatment decisions and must be considered in translational models. PJIs are classified as early (up to 3 months postoperatively), delayed (3 to 24 months postoperatively), and late (more than 24 months postoperatively),^82,83^ with time points chosen to align with typical pathologies. Early PJI development is typically an acute postsurgical infection. Delayed infection can follow chronic latency, with low level or undetectable infection over months or years, followed by a seemingly spontaneous infection. Infections can often be culture-negative, where viable causative organisms are not isolated from either blood samples or local swabs.^80,84^ This late infection indicates either chronic latency and mature biofilm, or a new, hematological source, and results in elevated failure rates.^85,86^ Therefore, in vivo models should attempt to accurately reflect these clinical stages of biofilm maturation in a predictable and reproducible manner.

## Treatment for Prosthetic Joint Infection

Regardless of clinical biofilm stage, surgical intervention in the form of débridement with antibiotics, irrigation, and implant retention (DAIR) is commonly the first course of action but has poor outcomes for entrenched infections ^87,88^. The physical removal of biofilm through surgical intervention and subsequent antimicrobial treatment of the planktonic shroud supports the final eradication of infection by the immune system, and cytotoxic levels of antibiotics must be avoided. The failure of DAIR is often due to the limitations of pulse lavage to completely remove biofilm from the retained implant.^89^ Decontamination of implants by autoclave is used midsurgery with some success; however, even a sterile physical biofilm matrix supports reinfection, and persister cells resist heat inactivation surprisingly well.^90–92^ Implant retention is preferred if feasible because of recovery time, morbidity, and overall cost.^93^

Local administration of antibiotic cocktails or slow-release beads may follow DAIR but can create a cytotoxic environment and impede the innate immune response when it is most needed.^36^ Final eradication of bacteria requires the immune system. Neutrophils can respond to a tissue infection within an hour, but they are much slower to home to abiotic surfaces, which delays the functional response to hardware infections.^4^ High antibiotic concentrations and other, more aggressive disinfection reagents can create zones of devitalized tissue in addition to the local necrosis expected by surgical intervention. Together, these increase susceptibility to secondary infection.^94,95^ With the passage of time and with each unsuccessful débridement,^96,97^ hardware removal becomes more necessary.^88,90,98^ This can be completed as a single procedure; however, a two-step procedure enables prosthesis-free antibiotic treatment and tissue regeneration for weeks or months before implant replacement. Failing these, the repercussions become increasingly dire, requiring months or years of antibiotic therapy and can lead to amputation and/or premature death.^48^ For these reasons, DAIR is often not recommended after just 3 weeks have passed since development of symptoms.^99,100^

Treatment of PJI can easily be delayed beyond this 3-week guidance because of gradual development of symptoms belying the urgency of the condition and the difficulty confirming the presence of infection and isolating causative organisms from infected joints. Reluctance to commit limited surgical resources to a professionally and personally demanding intervention based on an unclear diagnosis is understandable and more widespread than it may seem.^101,102^ Clinical microbiological results can require 2 weeks to return reliable negative results in growth-based assays, but this method is becoming rare because of timing and inaccuracy—some report as much as 56% false-negative output for preoperative synovial fluid aspirate,^60,103,104^ driving a trend toward rapid molecular methods such as alpha-defensin, each with their own weaknesses.^105^ Even in the presence of a positive culture, notable challenges such as the heterogeneous growth rates of biofilm bacteria play an important role in determining an effective treatment.^88^ Slower growing species can be overwhelmed in planktonic laboratory culture systems, leading to treatments targeting the faster growing CFUs over the slower growing cells, which are more likely to contribute to the underlying chronic condition.^100,106,107^ Suboptimal antibiotic selection can be more damaging than suboptimal treatment duration, resulting in culture negative infection over indefinite periods.^108^ This has led to recommendations that at least 5 biopsies from separate locations should be taken for extended culture (which may take weeks), and that antibiotic treatment should be suspended prior to sample procurement, putting patient welfare at risk.^110^ Resistance testing of the planktonic isolate(s) follows to identify an appropriate antibiotic regimen, with the expectation that tolerance exists in any mature infection.^8^ PJIs are difficult to accurately diagnose and expensive to treat efficiently, making effective translational models of the most challenging conditions, culture-negative biofilm, important to consider.

## Critical Features of Effective Prosthetic Joint Infection Models

In this section, we outline three critical features of effective PJI models: minimization of the planktonic shroud, generation of the biofilm phenotype, and quantification of the relevant infection. We believe the most effective path to a biofilm-based infection is inoculation with a mature biofilm-derived inoculum. The biofilm-derived inoculum must be verified by confirming the presence of the characteristic three-dimensional structure, or depth, of a biofilm. Finally, quantification of the infection as an end point, either directly or indirectly, is critical for clinically translatable results. These features must be considered when designing effective experiments that most accurately and precisely depict clinically relevant disease.

Quantitation of inoculum is a basic requirement for reproducibility in microbiology,^111^ and the nature of that inoculum is clinically relevant. As few as 100 CFUs of biofilm bacteria can initiate a PJI, validating the rejection of the 10^5^ rule of thumb for infection by Zimmerli et al. and reinforcing the idea that biofilm inoculation is the likely cause of dSSIs.^52,112^ Researchers using pure culture to inoculate animal models of infection must apply more planktonic CFUs to initiate a chronic condition. This results in unnatural amplification of the innate immune response and may require other modifications such as balancing mortality against morbidity to accurately represent the chronic disease.^113^ The convenience of using planktonic inoculation is associated with notable and important confounding effects. Bacteria living in biofilms have different gene expression and metabolic indicators in addition to their association with extracellular polymeric substances, all of which persist after inoculation, affecting the disease progression, response, and outcomes.^114–116^

The provision of a verified and quantifiable biofilm inoculum is challenging but has critical translational value. Physical attachment to hardware is not implied by proximity alone. Loosely associated purulence can be a significant confounder, because it may contain CFUs that are not biofilm derived but may be counted as such depending on upstream methods and the model in question. The application of methods that verify three-dimensional aggregation (3DA) of biofilm, such as electron or confocal microscopy is important for late-stage biofilm, but any method that can confirm self-aggregation is reasonable.^117^ We propose that because 3DA or gradient formation is a defining characteristic of the in vivo biofilm, visual confirmation of this structure is needed, in the absence of direct methods of measurement, to validate any model of chronic hardware infection.

Quantification of an in vivo biofilm by researchers is increasing in frequency but remains unstandardized. We recommend focusing efforts on quantifying the specific phase of biofilm under investigation. Attached CFU enumeration is a clear measurement of the attachment phase. Confounders such as purulent deposits that can harbor planktonic CFUs must be rinsed off and enumerated separately. Attachment data are typically collected by disruption of the cells with sonication and manual colony counting, assuming that all viable cells are separated, and each isolated cell grows to form a visible colony. Accumulation can be measured using total biofilm coverage area,^47^ but these methods remain subject to confounding if local acute infection cannot be separated from the data. The 3DA in the biofilm coverage area indicates the extent to which maturation is underway and, taken with attachment and accumulation data, can provide strong quantitative evidence of biofilm infection. Practically, performing both 3DA and CFU enumeration on the same implant is difficult because most imaging methods require destructive processing or time-consuming imaging that affects subsequent bacterial viability. In large animal models, having separate or separable implants in one subject for different measures of biofilm burden can ameliorate this problem, but this is more difficult in smaller animals.

Ultimately, biofilm cells per unit area is the measurement of interest; however, biofilm is rarely observed experimentally in the absence of confounding CFUs from the local planktonic shroud. Notably, CFUs shed into this shroud may still exhibit biofilm metabolic characteristics such as growth heterogeneity and tolerance, further complicating any strategies to enumerate infection. Bioluminescence produced by specific bacterial strains (such as *S aureus* XEN36) is a popular and convenient surrogate measure to quantify bacteria in an infection in small animal studies.^118^ Biophotonic imaging is a pseudoquantitative indicator of metabolic activity that often does not penetrate cortical bone or thick tissue and, thus, has limited applicability in quantitative biofilm modeling,.^119,120^ Further to this, biophotonic imaging imparts little information about the degree of attachment, accumulation, or maturation and provides limited information on the location.^121^ So, although direct quantitation of biofilm-specific CFUs in each state is emphatically recommended, this is often not possible because of its proximity to more easily quantifiable and metabolically active bioburden.

Indirect quantification methods, such as bone erosion or tissue damage, have clinical relevance. Culture-negative infections are capable of damaging tissue and eroding bone over time,^122^ and these events are indicative of strong animal models of chronic infection. However, planktonic infection can also cause tissue damage, and our recommendation is that more quantification of inoculation and final bioburden is required for results to be translated clinically if we are to see biofilm-based research as an improvement over historical methods.

## Current In Vivo Models of Prosthetic Joint Infection

To support our assertions that limitations on the adoption of appropriate terminology could be associated with clinically limited in vivo models, we analyzed a subset of animal models of prosthetic infection using the Medical Subjects Heading (MeSH) Index. It is manually curated and, although limited, serves as an objective subset of the area of interest, with Prosthetic Infection coded in as of 1993. In total, we assessed 109 relevant manuscripts of a total 122, from 2010 to 2019, after removal of redundant articles and reviews using the following search terms: “Prosthesis-Related Infections”[MeSH] AND “Models, Animal”[MeSH] not review[publication type]. These articles were assessed according to the criteria proposed earlier for features of effective PJI models: minimization of the planktonic shroud, generation of the biofilm phenotype, and quantification of the relevant infection.

**Figure 3:**
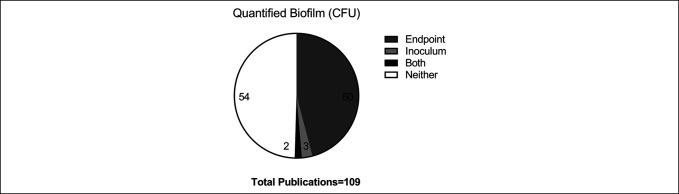
Pie chart showing quantification of biofilm in animal models of prosthesis-related infection published from 2010 to 2019. One hundred nine articles identified as animal models of prosthesis-related infection by infection by MeSH search from search from 2010 to 2019 were reviewed in depth. These were reviewed and coded for their use of biofilm as inocula and end points.

Our investigation indicated that nearly 50% of animal models of prosthetic infection did not report a quantified biofilm inoculum or final bioburden in their studies (Figure 3), which would normally be a requirement of animal models of infection.

**Figure 4:**
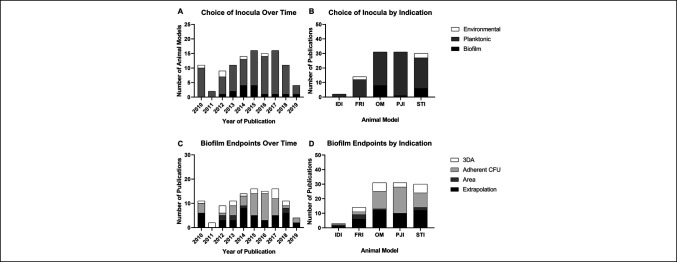
Bar charts showing the use of biofilm inocula and end points in different types of animal models of infection from 2010 to 2019. Animal models of prosthesis infection were identified from the MeSH Index from 2010 to 2019 (n = 109). Models were manually categorized as indwelling device infection (IDI), fracture-related infection (FRI), osteomyelitis (OM), prosthetic joint infection (PJI), and soft tissue infection (STI). Choices of inocula were identified as planktonic or pure culture inoculation, environmental or passive inoculation, and inoculation by preformed biofilm. **A**, Methods used to inoculate the prosthetic by year of publication. **B**, Methods used to inoculate the prosthetic by medical indication under investigation. Notably, OM and STI account for all but one instance of inoculation using biofilm. **C**, Methods used to verify the presence of biofilm at data collection by year of publication. Adherent CFUs indicate bacteria is colocalized and not necessarily attached. Enumeration of adherent CFUs is most common, followed by extrapolation based on infection duration in proximity to prosthesis. Direct verification of three-dimensional aggregation (3DA) or total biofilm coverage area is rare. **D**, Methods used to verify the presence of biofilm presence at data collection by medical indication under investigation. There is no clear preference of methods by indication in this data set.

We found that inoculation of animal models with biofilm according to these criteria are uncommon, and no indication of increased use over time was found (Figure 4, A). Of the 15 studies that reported inoculation of in vivo models with biofilm, only three used biofilm cultured in excess of 24 hours, which is typically believed to be sufficient for maturation of biofilm.^123,124,125,126^ Of the remaining 12 models, 5 allowed the initial planktonic inoculum less than 1 hour to adhere, leaving considerable room to question the presence of biofilm. Most importantly, all but one of these examples came from soft tissue infection or osteomyelitis models, with the more relevant models of fracture-related infection and prosthetic infection virtually devoid of biofilm-based inoculation (Figure 4, B).

Verification that biofilm is present in the animal model was more encouraging, with 18 of 109 models using either scanning electron microscopy or 3-dimensional fluorescence microscopy to identify the appropriate 3DA. Although a clear trend over time was not found in the choice of analysis (Figure 4, C), the identification of 3DA as an end point is relatively consistent across indications. Still, a significant proportion of studies extrapolated the presence of biofilm from the presence of infection over time. Ultimately, although verification of biofilm phenotype was attempted in greater than 50% of the articles reviewed, most of the verification methods chosen are not conclusive, stand-alone methodologies.

Finally, although attempts to quantify the biofilm component of an infection may be on the rise, the methods used are often indirect and provide data on closely associated or localized CFUs, not biofilm. Arguably, in many cases, this was simply not possible given the tools available and the state of model development. For the purposes of this review, we comment on the challenge and encourage researchers to qualify their data as indicative of infection and not necessarily a mature biofilm in the absence of data to support the claim. Notably, enumeration of adherent CFU has emerged as the most common method for quantification of hardware-bound biofilm across most prosthesis-related infections (Figure 4, D). Some method of quantification of the overall infection was used in most cases, including CFU enumeration from soft tissue or bone, histologic assessment, positive blood cultures, or live/dead staining of representative areas. These methods all provide critical information, but they do not specifically quantify biofilm.

## Summary

The economic burden of PJI revision in the United States is projected to exceed $1.6 billion by 2020,^127,128^ which is a single indication of chronic infection. With nearly half a million FDA-registered devices of increasing complexity, and nearly more 4000 in activate clinical trials at the time of submission, expecting a decline in procedures is not reasonable. The elective application of implantable technology is expected to increase the incidence of dSSIs as healthcare consumerism continues its upward trend.^129^ Because the patient-associated and economic incentives for pursuing this area of research are plentiful, this research should be done with rapidly translatable outcomes in mind.

PJI is an excellent model for chronic hardware infection because of convenient anatomical characteristics that isolate it from systemic influences and limits the spread of infection to unrelated tissues. Chronic hardware-related musculoskeletal infection is historically an orthopaedic issue, but recent advances in tissue engineering and medical device complexity have brought the challenge of dSSI to new frontiers. We have demonstrated that biofilm terminology is rapidly being adopted by groups less likely to use historic terms such as prosthetic or periprosthetic infection, but lags in specialties such as orthopaedics where highly influential translational research occurs. The potential exists for this to negatively affect study design, and discussion is warranted.

A substantial amount of work remains for PJI research to produce paradigm-shifting clinical advances. For regulators, the challenge is clear from the dearth of tools to effectively differentiate chronic/biofilm infection from acute/planktonic infection. The appropriate terminology has not penetrated important areas of research, and evidentiary standards have not evolved, contributing to regulatory paralysis in a stagnant cycle. For researchers, detecting these metabolically inactive bacteria in the absence of rapidly growing acute infection is critically important to clinical translation because the latter is not what drives recalcitrant infection. This must be an expectation for animal models. For reviewers, the challenge of regulatory and clinical definitions cannot lower the bar for evidence.

We propose using biofilm definitions consistent with clinical observations to facilitate overcoming these challenges and have provided a mapping strategy for biofilm stages, clinical presentation, and key data. In summary, we propose the following:

1. Regulators must define clinically relevant terminology that can be adopted by researchers. Adherence, accumulation, and maturation determine the recalcitrance of the infection, link clinical indication to disease models through microbiology, and could provide much-needed cross-functional clarity and consistency.
2. Reviewers should require critical biofilm feature verification from in vivo models. Specific qualification and quantification of biofilm is more important, not less, than in historic pure culture models and cannot be assumed from one-dimensional CFU data.
3. Researchers need to model the disease accurately and reproducibly by inoculation with representative biofilm and selecting for tolerant localized infections representative of clinically culture-negative infections.
